# Supplementary material for: Are carbon emissions trading and green financial instruments synergistic? -Comprehensive quantitative research based on content analysis
Source: PLoS One. 2024 Mar 7;19(3):e0298601. doi: 10.1371/journal.pone.0298601 (PMC10919601; doi:10.1371/journal.pone.0298601)
Supplement: S1 Data — (ZIP) [file pone.0298601.s001.zip › Data/地级市Z_F工作报告.zip!/╜¡╦╒/╒≥╜¡╩╨/└┤╫╘╣1⁄2╓┌║┼í╛┬φ┐╦╩2╛▌═°í┐_2014.docx]

各位代表：

　　现在，我代表镇江市人民政府，向大会作工作报告，请予审议，并请各位政协委员提出意见。

　　2013年工作回顾

　　刚刚过去的一年，是镇江在现代化建设征程上奋力前行的一年。在省委、省政府和市委的正确领导下，我们紧紧依靠全市人民，全面贯彻党的十八大、十八届一中、二中、三中全会和习近平总书记系列重要讲话精神，扎实推进“八项工程”（1），全力实施“四大行动”（2），取得了经济社会发展的新成就，较好完成了市七届人大二次会议确定的各项任务。预计全年实现地区生产总值2950亿元，增长12%；公共财政预算收入254.5亿元，增长18.1%；固定资产投资1777.2亿元，增长22.1%；城镇居民人均可支配收入、农民人均纯收入分别达33050元、16260元，增长10%和12%。

　　一年来，围绕全市现代化建设的总体要求，重点开展了八个方面工作：

　　一、抓项目、建园区，产业“三集”（3）有效推进。在推进重大产业项目上，通过领导挂钩、每月调度、现场观摩等方式，不断浓厚项目建设氛围。200个重点产业项目完成投资658亿元；举办140余场专题招商活动，新批外资项目162个，预计实际利用外资30.7亿美元；新增到位国资147.7亿元；实际利用市外民资535.7亿元。丹阳市鱼跃医疗设备、国际眼镜城等一批超50亿元项目加快推进；句容市总投资超百亿元的雨润欢乐城、超40亿元的轨道交通等项目顺利签约；扬中市中海粮油加工产业园等5个百亿元项目成功引进。在推进产业集中集聚集约发展上，坚持每月调度、现场督查，三大类80个产业园区规划基本完成，20个先进制造业特色园区实现应税销售1230.9亿元，占全市的37.6%；30个现代服务业集聚区实现营业收入910亿元，占全市的48%；30个现代农业产业园区新增高效农业面积8万亩，占全市的35.6%，新增2个达到省级标准的农业园区。中瑞生态产业园（4）加快推进，镇江生态文明先行区开始启动，对台合作十大载体着手建设，海峡两岸新材料产业合作示范区获国家批准。在全力服务企业发展上，每月召开企业家早餐会，及时出台促进小微企业健康发展相关政策，深入企业开展“帮困解难服务月”活动，有效解决各类问题。全年净增定报企业324家，新增年销售超百亿企业2家、总数达9家。

　　二、转方式、调结构，创新发展步伐加快。农作物优良品种覆盖率达95%，粮食生产实现“十连增”，“戴庄经验”（5）全省推广。编制航空航天产业等发展规划，新兴产业呈现集聚发展态势，实现销售3002亿元，增长33.2%；完成技改投资241.9亿元，增长25.9%。服务业增加值占GDP比重达43.2%，同比提高1个百分点。全市旅游业发展总体规划编制完成，镇江电子口岸服务平台投入使用，惠龙港物流交易额超500亿元，镇江“好产品网上展销馆”上线运行；社会融资规模达874亿元，其中直接融资383亿元，成立市金融产业发展有限公司。国家创新型城市试点和国家知识产权示范市建设扎实开展，新增9家博士后科研工作站和4个省级以上科技孵化器；全社会研发经费支出占GDP比重达2.45%，高新技术产业产值占规模以上工业产值比重达46.8%，保持全省第一，连续第九次荣获“全国科技进步先进市”称号。国家“千人计划”（6）专家总数突破50人、省“双创”计划（7）人才（团队）达200人；成功举办首届技能人才博览会。

　　三、重统筹、优功能，城乡面貌显著改变。主城区东吴路绿地广场、官塘桥路快速化改造、跑马山公园等40项城建重点工程如期完成。312国道南移、五凤口高架等骨干交通项目加快推进，京沪高铁沿线环境整治和绿化获省一等奖。“7+1”旧城区城中村改造（8）征收拆迁基本完成。高校园区完成部分基础设施建设，镇江技师学院主体竣工，市委党校、镇江高专和共享区体育馆开工建设。深入开展城市环境综合整治，“美丽城市”市长月度督查活动取得积极成效。丹阳市滨江新城总体发展规划通过专家论证，练湖水城建设及西城区等片区改造全面拉开；句容市北部新城建设全面启动，宁杭高铁句容西站投入运营，宁句城际轨道交通签订合作框架协议；扬中市完成奥体中心主体建设，长江三桥建设进展顺利。全市“三新”建设（9）深入推进，新建农民安置房21.8万平方米。村庄环境整治全面完成，顺利通过省级验收。古运河风光带加快建设，江心洲水毁修复工程顺利推进，抗旱救灾工作有效开展。

　　四、抓整治、促提升，生态建设成效明显。率先编制实施主体功能区规划，划定生态红线。扎实推进国家低碳城市试点工作，低碳九大行动（10）深入实施，官塘新城成为亚太经合组织低碳示范城镇试点。国家森林城市40项创建指标全部达标，新增绿化造林9.7万亩。东部地区异味治理和韦岗地区大气环境整治取得阶段性成果，“蓝天工程”（11）83个项目全面完成，秸秆禁烧工作全省领先。完成国家级废弃矿山地质环境治理一期工程。关闭化工企业61家，淘汰落后产能企业35家。太湖流域水污染治理126项工程扎实推进，市区水环境综合整治深入开展，地表水好于III类水质的比例达66.1%。生态文明建设工程监测指数列全省首位，成为全国生态文明建设试点城市，顺利通过中国人居环境奖专家组考核验收。

　　五、顺民意、惠民生，群众生活持续改善。“百村万户”双达标行动（12）深入开展，50%的农村低收入户和经济薄弱村达标。扶持创业1.2万人，新增城镇就业7.2万人，转移农村劳动力3.2万人，城镇登记失业率为2.3%。社会保障机制不断完善，城乡基本保障覆盖率达97.5%，城乡低保标准实现一体化。物价水平总体稳定，发放临时价格补贴1022万元，惠及5万多名困难群众；新增平价商店31家。文明城市创建综合测评取得近年来最好成绩。成功举办“长江国际音乐节”，新增8处全国重点文物保护单位，建成市民公共文化广场260个。企业职工文化建设经验全省推广。“赛珍珠文化交流”活动富有特色，大韩民国临时政府史料馆建成开馆。率先推进苏南教育现代化示范区建设，扎实开展国家学前教育体制改革试点，稳步推进校车公交化改革，创办镇江第一外国语学校、恢复崇实女子中学，东方技校投入运行，镇江高专通过省级人才培养工作评估，江苏大学、江苏科技大学等高校为地方发展作出积极贡献。“健康镇江行动”（13）深入实施，公立医院改革不断深化，市二院、市精神卫生中心顺利搬迁，318家村卫生室标准化建设全面完成。市体育会展中心建成开放。成功举办直通巴黎男子乒乓球选拔赛、舜天中国足球超级联赛，城市生活融入新元素。

　　六、打基础、强基层，社会活力不断增强。圆满完成村居委员会换届选举工作，城乡和谐社区达标率分别达85.6%和88.3%。“平安镇江”、“法治镇江”建设和“六五普法”工作扎实推进，社会管理不断加强，信访形势总体平稳，再度获评全国社会治安综合治理优秀地市，荣获“长安杯”。第三次经济普查扎实开展。全国双拥模范城“七创”工作通过中期评估。建立健全应急管理体系，“物资库”、“专家库”建设和实战型应急演练取得积极成效。有效应对人感染H7N9禽流感疫情。安全生产事故起数和死亡人数连续12年“双下降”，食品药品和产品质量安全责任得到有效落实。审计、人口计生、侨务、民族宗教、机关事务等工作取得新成绩，编制、档案、地方志、防震、供销、科普、老龄、妇女儿童、关心下一代、残疾人、慈善和红十字等事业取得新进展，国家安全、国防教育、人民武装、人民防空、援藏援疆等工作进一步加强。

　　七、抢机遇、创特色，各地发展亮点纷呈。丹阳市规划建设“6+1”重点产业园区（14），新增上市企业2家，创成全省首家军民结合产业示范基地。句容市抢抓宁镇扬同城化机遇，城乡发展活力不断增强，现代农业、生态建设走在全省前列。扬中市成功举办第八届省园博会，加快推进产城融合，充分展示“小城办大事”的魄力。丹徒区积极呼应主城南移，以北汽项目为带动，大项目建设、高校园区建设和生态建设成效明显。京口区坚持现代服务业和先进制造业“双轮驱动”，爱励铝业、财富广场等一批重大项目加快推进。润州区一手抓国家级高新区创建，一手抓现代服务业发展，区域经济整体水平明显提升。镇江新区以跻身国家级开发区第一方阵为目标，狠抓项目、载体和人才建设，综合发展评价指数由第24位上升至18位。“三山”景区扎扎实实做规划、打基础、上项目，长江路景观提升等工程顺利完成，旅游综合收入实现翻番。

　　八、转作风、提效能，自身建设全面加强。坚持集思广益。重大行政决策充分听取人大、政协、民主党派、工商联、无党派人士、人民团体和专家学者、老同志、网民代表、广大市民等方方面面的意见和建议，并自觉接受监督，不断推进决策的民主化、科学化。坚持依法行政。完善规范性文件、重大决策合法性审查机制，健全政务公开、定期新闻发布制度，推进行政权力网上公开透明运行，在全省率先完成法治政府建设阶段性工作目标。政府系统承办人大代表建议169件，满意率达100%；办理政协提案383件，满意和基本满意率达100%。坚持例会制度。每月召开一次市长工作会议、两次政府常务会议、两次规委会，举办一次政府系统领导干部集中学习会。一年来，规委会共对300多个项目进行了严格审查，保证了项目质量。坚持转变作风。严格执行中央“八项规定”和省、市委“十项规定”（15），大力压缩一般性支出，“三公”经费（16）明显下降；以市政府名义下发文件同比减少37.6%。立足解决问题，深入基层一线，扎实开展“三解三促”活动（17）；以“加强纪律性、增强执行力”为主题，在全省率先开展公务员集中军训，全面提升了公务人员的精气神。坚持提升效能。加强服务型政府建设，行政审批事项压缩25.3%，成立市政务服务管理办公室、市公共资源交易中心，承诺件办理时限由14.6个工作日压缩到4.9个。

　　需要向大会报告的是，受宏观环境和政策变化影响，市七届人大二次会议确定的个别指标未能完成；少数项目也未能按计划实施，对此都按照投资总额不变、项目个数不减的要求，及时替换了新项目。在今后的工作中，我们将努力加以防止，确保人代会确定的各项任务顺利完成。

　　各位代表！回顾去年的工作，我们最深刻的体会是：作决策、抓发展、办事情，一定要以镇江人民感到高兴、满意和自豪为出发点和落脚点，一定要用踏踏实实做事、真心实意为民来赢得全市人民的信赖、支持和认可。一年来，我们抓了主体功能区规划、产业“三集”发展、低碳城市建设、北汽项目落户等打基础、利长远的大事，做了中山西路改造、南门大街整治出新等改变城市面貌的实事，办了智能公交、南山绿道、公共自行车投放等受到普遍认可的好事。实践证明，人民与党委、政府良性互动谋发展、同心协力干事业，是推进现代化建设的力量源泉，更是今后必须长期坚持和弘扬的宝贵财富。在此，我代表市人民政府，向全市人民表示崇高的敬意和衷心的感谢！向各位人大代表、政协委员，向市各民主党派、工商联、无党派人士、人民团体和各界人士，向驻镇人民解放军、武警官兵和人民警察，向垂直部门和驻镇单位，以及离退休老同志和所有关心支持镇江发展的海内外朋友们，表示衷心的感谢！

　　我们也清醒地认识到，发展中还存在着一些问题：产业结构性矛盾仍然突出，自主创新能力不强，服务业占比不高，节能减排任务艰巨；优质大项目仍然缺乏，经济增长的后劲和动力不强，发展质量和效益有待进一步提高；城乡规划、建设和管理仍存在一些不足，人民群众对生态环境问题反映强烈；城乡居民收入增长不快，民生改善还需继续加大力度；机关作风、服务效能还要进一步改进和提升。对这些问题，我们将高度重视，扎实加以解决。

　　2014年主要任务

　　今年，是贯彻落实党的十八届三中全会精神的开局之年，是全面完成“十二五”规划目标任务的关键之年。世界经济将延续缓慢复苏态势，国内经济长期向好的基本面没有改变，镇江正迎来苏南现代化示范区建设、省生态文明建设综合改革试点、宁镇扬同城化、上海自贸区建设等重大机遇。我们完全有基础、有条件、有能力开创全面深化改革的新局面，迈出现代化建设的新步伐！

　　按照市委六届七次全会的部署，政府工作的总体要求是：深入贯彻党的十八届三中全会、中央经济工作会议和省委十二届六次全会、全省经济工作会议精神，以苏南现代化示范区建设为统领，以生态文明建设综合改革为主抓手，坚持稳中奋进，着力改革创新，咬定“六大任务”，创造“镇江特色”，全面增强综合实力，全面提升民生福祉，全面加快现代化进程。

　　今年经济社会发展的主要预期目标是：地区生产总值增长10%；公共财政预算收入增长12%；固定资产投资增长18%；社会消费品零售总额增长12%；产业类实际利用外资（不含房地产）增长8%；全社会研发经费支出占GDP比重达2.55%；城镇居民人均可支配收入增长10%；农民人均纯收入增长11%；城镇登记失业率控制在3%以内；全面完成省下达的节能减排约束性指标。

　　做好今年的工作，我们将立足“稳中奋进、改革创新”这一主基调，积极把握国内外形势的新变化，充分认识镇江发展的新特征，正确处理“好”与“快”、“质”与“量”的关系，做到既务实可行，又奋力作为；既保持有效增长，又为深化改革、调整结构留出空间。在工作安排上，将着重抓好六大任务：

　　一、突出转型升级，加快构建现代产业体系

　　按照市委全会提出的“差别化转型升级”的要求，全力以赴推进200个以上重点产业项目，确保年度投资670亿元以上。

　　推动先进制造业高端发展。强化产业政策的配套联动，推进47个固定资产投资10亿元以上的制造业项目，当年完成投资200亿元以上。重点发展优势主导产业。高端装备制造业围绕汽车及零部件、航空航天、海工配套等，加快推进北汽、中陆航星等20个在建在手重点项目，确保当年完成投资95亿元。新材料产业突出高性能合金材料、碳纤维及其复合材料、新型化工材料等，加快推进恒神碳纤维、爱励高档铝合金板等12个在建在手重点项目，确保当年完成投资48亿元。培育发展新兴产业。围绕生物技术与新医药、新能源、智能电网、新一代信息技术，加快推进大全智能电器、金泰生物等项目建设，集聚关联企业，完善产业链条。推进传统产业提档升级。运用低碳技术和信息化技术，改造提升机械、化工、轻工等传统产业，完成技改投资突破300亿元。淘汰落后产能。实施20个重点项目，关闭小化工企业50家以上，“五小”企业（18）整治率达70%以上。按照“四个一律”（19）的要求，制定产业发展“负面清单”（20），对新上项目严格把关。

　　推动现代服务业提速发展。推进57个10亿元以上服务业项目（不含房地产），当年完成投资289亿元，力争服务业增加值占GDP比重达44.5%，新增1-2家省级现代服务业集聚区。加快发展物流业，实现营业收入625亿元。着力整合岸线、港口、道口等资源，加快推进扬中夹江航道整治工程，建设海事监管现代化镇江示范区，推进镇江进口商品集散中心、丹阳智能云仓储中心等项目。提升发展旅游业，实现综合收入600亿元。着力实施“三山”5A景区提升工程，推进茅山争创国家5A级旅游景区（21），加快丹徒世业洲、句容茅山湖、丹阳水晶山省级旅游度假区建设，高水平建设国家智慧旅游服务中心，开通旅游公交专线，实施旅游惠民“一卡通”。加速发展文化产业，实现营业收入520亿元。着力建设海峡两岸文创园、国家数字出版基地镇江园区、十里长山文化产业园等项目，支持文艺、演艺类作品创作，促进创意设计、广告会展等行业发展。积极发展平台经济，着力培育电子商务、云计算、物联网、移动互联网等新兴业态，积极支持和推动一批平台型企业发展，培植和引进一批与之相配套的第三方服务机构。

　　推动现代农业高效发展。在确保粮食安全的基础上，深化农业结构调整，重点发展优质粮油、特色园艺、特种养殖、高效林业、休闲农业五大特色产业，强力推进森禾花卉、山水湾农业生态园等项目，力争吸引“三资”投入农业50亿元以上。深化农村改革，加快构建新型农业经营体系，大力推广“戴庄经验”，新增达到省级以上标准的农业园区4家、省级农业龙头企业5家。支持句容、丹阳创建国家级现代农业示范区。继续加强农田水利建设，完善河塘沟渠灌溉体系，净化农产品产地水土环境，新增高标准农田7.5万亩。

　　加快推进产业“三集”。坚持分类指导、重点突破、创造特色，扎实开展“园区建设提升年”活动，推动80个特色产业园区在规划布局、完善功能、项目进园、差别考核等方面做实做优，实现先进制造业特色园区固定资产投资30亿元以上项目、现代服务业集聚区20亿元以上项目、现代农业产业园区1亿元以上项目全覆盖。

　　全面扩大对外开放。以中瑞生态产业园为载体，积极接轨中国（上海）自由贸易试验区，在政策创新、组织架构、运作方式等方面先试先行、创出特色，力争成为中瑞自由贸易示范区，构建对瑞士乃至欧盟的合作交流平台。加快现代物流和服务贸易发展，推动出口加工区转型升级，争取创成国家级综合保税区。积极推进生态文明先行区开展国际合作。加快海峡两岸合作十大载体建设，打造对台合作“第一福地”。加大产业项目招引力度，力争各辖市区和镇江新区新引进总投资5亿美元以上项目全覆盖；支持丹阳创建国家级经济技术开发区。不断扩大对外贸易，鼓励有实力的企业“走出去”。

　　二、强化规划引领，大力推进生态示范区建设

　　以天蓝、地绿、水净为目标，推进低碳建设，加快生态修复，实施生态环境建设项目89个、年度完成投资101亿元。

　　加强综合整治。整治“两个片区”（22）。用3年时间，对市区东部谏壁地区和西南韦岗片区实施环境整治，下大力气解决人民群众反映强烈的环境问题。从今年起，在谏壁地区实施索普集团、谏壁电厂污染治理等26项企业治污项目，有序实施相关区域居民搬迁；在西南片区有序关停小矿山、小水泥、小石灰窑、小化工企业，加快推进生态修复和居民搬迁等工作。整治“一湖九河”（23）。对金山湖和市区古运河、运粮河等9条河道及支流开展为期3年的水环境综合整治，通过控源截污、清淤疏浚、环境整治、引水活水、生态修复和景观提升，实现“水清岸绿、鱼虾洄游、环境优美”的目标。整治大气污染。强化问责机制和应急预案，有力推进工业废气和粉尘治理、秸秆禁烧和综合利用、机动车尾气治理、施工扬尘污染防治等工作，确保PM2.5（24）浓度到2017年下降20%左右。

　　建设低碳城市。大力推进“碳平台、碳峰值、碳评估、碳考核”创新，在国内率先建成和运营城市碳排放核算与管理云平台。启动实施一批低碳示范项目，加快官塘低碳新城、低碳高校园区、科技新城低碳园区等示范区建设，力争率先创成全国低碳示范城市。构建循环经济体系，加快镇江新区国家级园区循环化试点，鼓励有条件的开发园区争创国家级生态工业园、循环经济产业园。

　　优化环境质量。围绕“三山”5A景区提升，完成景区西入口、金山湖北岸风光带、长江湿地保护等项目建设。建成南山北入口和南山绿道二期工程，推进市区绿道与各辖市绿道联网成环，逐步构建大生态走廊。实施国家级废弃矿山地质环境治理二期、句容赤山湖湿地公园等生态修复工程。开展碳汇林建设，新增绿化造林3万亩，林木覆盖率达26%以上，创成国家森林城市。继续开展山体整治，新建沙山、四平山等5座山体公园。切实加强水源地保护，抓好太湖流域、长江水污染防治。严厉打击各类非法偷排行为。

　　三、突出高雅品质，精心打造山水花园城市

　　以城市国际化、乡村田园化、城乡发展一体化为方向，扎实推进城乡建设，完成年度投资990亿元，其中市区690亿元，着力彰显生态、文化、宜居的品质和特色。

　　提升主城品质。坚持以规划为龙头，完成城市总体规划修编，推进城市设计全覆盖。提升长江路、建成京江路，形成南北两条串连“三山”的滨江风光带。实施中山东路、解放路街景整治和亮化，建成苏宁广场裙楼和财富广场。加快推进“7+1”地块项目建设。启动鼓楼岗、和平路西等片区改造，完成征收拆迁280万平方米。整治老住宅小区25万平方米，实施梦溪园等片区空斗墙改造。扎实推进南徐新城、官塘新城等新片区开发。高校园区全面建设共享区和配套区，江苏科技大学新校区、江苏大学京江学院部分建筑主体封顶，镇江高专新校区主体建筑封顶，省交通技师学院全部主体封顶。继续完善城市功能，科学统筹施工安排，市区新改建道路21条；征润洲取水口加快实施深泓取水工程，大港水厂完成主体工程，江心洲新自来水厂建成供水；加快智慧城市建设，推进20个市政公共场所免费无线上网。在城市各重要节点设置中外文标识，增添国际化元素。

　　统筹区域发展。坚持以人为核心的新型城镇化，推动主城区、各辖市城区、重点中心镇、一般建制镇合理分工、功能互补、协同发展。丹阳加快建设滨江新城、练湖水城，句容加大旧城改造和北部新城开发，扬中加速构建 “一主一副”新架构。支持各重点中心镇成为农民转市民的主平台，引导一般建制镇因地制宜、特色发展。加快构建“大交通”格局，推进312国道南移，完成市区至扬中、开工市区至丹阳和句容的快速通道，完成茅以升大道主体工程，建成大路通用机场，启动连淮扬镇铁路、地铁1号线前期工作，开工建设沪宁高速句容北道口，力争开工建设宁句轨道交通S6线。推进公共设施向农村延伸，完成国省干线公路环境整治和绿化100公里，新改建农村公路300公里，镇村公交开通率达100%，加大燃气、污水处理等管网向乡村延伸的力度。加快推进城乡教育、医疗、社会保障等公共服务均等化。深化村庄环境整治，建成1个“美丽宜居小镇”、10个“美丽宜居村庄”，争创15个省三星级康居村。

　　全面提升城市管理水平。改革环卫作业考核办法，理清“干、查、督”各方职责，形成闭合循环运行机制。对151条主次干道建立“街长制”，延长夜间保洁时间，有效改变“脏乱差”现象。深入开展城市环境综合整治，集中对渣土车、人力客运三轮车、货运车和犬类管理等方面存在的问题开展专项整治，并建立长效管理机制。升级数字城管平台，加大动态监管力度。大力推进“全民城管”，全面落实“门前三包”责任制，调动广大市民共建美丽家园。争创“江苏省优秀管理城市”。

　　四、突出创新驱动，提升科技对转型升级的支撑作用

　　紧紧围绕全市主导产业、优势产业以及未来成长空间大的产业，实施科技创新项目35个，完成年度投资85亿元。

　　推进载体建设。加快建设镇江知识城、水科技产业园、中船海工配套产业园，全力争创国家级高新技术开发区。加强科技创新社区建设。全市新增孵化面积50万平方米以上，推动1-2家科技孵化器升格为国家级。加快丹阳军民结合产业示范区、句容健康科技产业园和宝华科技新城、扬中复旦产业园、丹徒氢能产业园发展，支持丹阳、扬中创建省级高新技术开发区。

　　狠抓企业创新。支持企业建设技术研究院、博士后科研工作站和重点实验室等研发机构，力争省级以上研发机构比重达41%，并切实提高其利用效率。深化企业与高校院所合作，定期举办产学研对接活动，培育协同创新示范企业100家，新增1-2个省级以上协同创新基地。

　　推动技术攻关。围绕战略性新兴产业组织科技攻关，突破一批核心应用技术。航空航天领域重点研发通用飞机整机制造技术；新材料领域重点研发高性能合金、碳纤维及其复合材料等关键技术；新能源领域重点研发风电设备、光伏、氢能等核心技术；高端装备制造领域重点研发智能电力电气和特种船舶相关技术。全年力争高新技术产业产值突破4100亿元，占规模以上工业产值比重超过48%。

　　加强人才建设。细化落实科技创新政策20条和人才管理改革试验区政策20条，培育引进创业创新领军人才150人，其中国家“千人计划”专家15人、省“双创计划”人才（团队）40人；加强人才培养，培育创业企业家100名、企业经营管理人才1000名，新增高技能人才10000名，进一步提高各类人才助推产业发展的成效。

　　强化科技服务。建设国家级知识产权服务集聚区，新引进2-3家知识产权中介服务机构，建成科技金融服务中心，推动设立1家以知识产权质押贷款为主业的小额贷款公司。设立市专利行政执法审理庭，加强专利保护。切实加大智力成果收益的保护力度，鼓励科技创业。

　　五、突出社会民生，不断改善人民生活

　　坚持把人民对美好生活的向往作为奋斗目标，按照“保基本、兜底线、促公平、可持续”的总体要求，实施民生改善项目57个，完成年度投资超50亿元。

　　提高保障水平。深入实施“城乡居民收入倍增计划”，有效增加居民收入。动态调整最低工资标准，推进企业工资集体协商。继续开展“百村万户”双达标行动，确保75%的农村低收入户、经济薄弱村达标。坚持以创业带就业，完善相关政策，切实加强培训，扶持创业7000人，新增城镇就业6.5万人，镇江籍高校毕业生年末就业率达95%。社会保障主要险种参保率稳定在98%以上，认真做好被征地农民进“城保”工作。新开工保障性住房8000套，提高住房公积金制度覆盖面。增强社会养老服务能力，新增机构养老床位1500张。提高低保标准和特困残疾人生活救助标准；进一步提升社会救助水平，大力发展慈善和红十字事业。

　　完善公共服务。新增新能源公交车150辆、公共自行车3000辆。创建市教育现代化先进镇（街道）7个、先进学校70所；新改扩建幼儿园18所；镇村学生接送实现公交化；筹建镇江现代装备高等职业技术学院，建立镇江市开放大学。积极推进“健康镇江”行动。加强医疗专业人才队伍建设。整合市妇幼保健院和市儿童医院。全力争创全国文明城市，广泛开展职工文化活动，不断提高公民道德素质、科学素养和全社会文明程度。加强文化遗产保护，加快建设“文化四馆”（25）和西津音乐厅，改造西津剧场，新建250个文化广场，力争通过省级公共文化服务体系示范区验收。新建市体育运动学校，新改建50个社区全民健身点，承办3项以上具有全国影响的体育活动。切实加强人口计生服务，做好单独二孩政策落地工作。继续加强公园、公厕、菜市场、停车场以及城市无障碍设施建设。建立市食品检测中心、农产品质量安全检测中心。支持各类网站规范运营、有序发展。

　　强化社会治理。立足加强基层基础建设，深化“网格+网络”管理，推进政社互动；完善“中心社区+基本社区”的社区组织管理框架，新建4家中心社区；对进入社区的各类事务和检查验收，分别试行代理制和准入制。开展行政服务标准化国家试点，建设集政务服务、公共资源交易、便民服务、行政效能监察和“12345服务热线”为一体的“市民之家”。完善征信体系，建设诚信社会。持续推进双拥“七创”和军民融合深度发展。建立健全安全生产“五大体系”（26），加大隐患排查整治力度，切实防范重特大事故发生。深化“平安镇江”、“法治镇江”建设，深入开展“六五”普法，有效化解各类矛盾，维护社会和谐稳定。

　　六、突出改革先行，充分释放发展活力

　　今年是改革之年。我们将认真贯彻十八届三中全会精神，按照市委关于全面深化改革的统一部署，具体探索十个方面的机制创新：

　　建立国有企业加快转型升级机制。改革市区投融资平台运营机制，推进资产证券化、股权多元化，扩大直接融资比例；推动国有企业完善现代企业制度，鼓励国有资本、集体资本、非公有资本等交叉持股，积极发展混合所有制经济。

　　建立主体功能区制度落实机制。推动主体功能区规划、城市总体规划和土地利用总体规划“三规合一”。全面实施产业准入、生态补偿和差别化考核等制度。

　　探索园区市场化运作机制。对部分有条件的园区，采取股权合作、引进第三方专业企业等托管模式，委托企业进行开发管理。

　　深化教育领域综合改革。取消重点班和重点校，加强薄弱学校建设，实行校长教师交流制度；建立完善学校自主管理、教师岗位管理和自主聘用等制度，扩大办学自主权；深入推进国家学前教育体制改革，扶持普惠性民办幼儿园发展；完善教育督导机制。

　　深化医药卫生体制改革。调整完善医疗保障政策，积极推进医保异地结算；深化公立医院和基层卫生改革国家试点，引进社会资本参与改制重组，有效化解公立医院债务；鼓励社会办医，对不同所有制医院给予同等国民待遇。

　　探索城乡发展一体化体制机制。稳步推进农村土地承包经营权确权登记试点，允许农民以承包经营权等资产性权属入股参与农业产业化经营，赋予农民更多财产权利。稳步推进农村集体资产社区股份制改革。

　　建立权力清单制度。制定政府各工作部门权力清单，公开行政权限、审批事项和办事流程。推进碳评、环评、能评、稳评与安评“五评合一”（27）。制定企业投资“负面清单”。

　　推进地方机构改革。实施市县两级政府机构改革，调整食药监、工商、质监管理体制，加强食品药品信息化监管；整合卫生、人口和计生部门。基本完成承担行政职能和从事生产经营活动的事业单位改革。

　　建立发展绩效评价机制。加大资源消耗、环境损害、生态效益、产能过剩、科技创新、安全生产、政府债务等指标的权重，更加重视劳动就业、居民收入、社会保障、人民健康状况。对适度开发区域和生态平衡区域拟定相关指标。

　　创新社会治理机制。以网格化管理、社会化服务为方向，健全基层综合服务管理平台。加快实施政社分开，推进社会组织明确权责、依法自治、发挥作用。大力推动政府购买服务。创新有效预防和化解社会矛盾的工作机制。

　　不断提升政府治理能力

　　新的形势和任务，对政府自身建设提出了更高要求。我们将扎实开展党的群众路线教育实践活动，把改革创新融入政府工作全过程，不断提升政府治理能力。

　　一是加快转变职能。紧紧围绕使市场在资源配置中起决定性作用和更好发挥政府作用，正确处理政府与市场、政府与社会的关系。该放的坚决放下去，认真做好国家和省已取消行政审批事项的落实工作，同时结合镇江实际，加大“减、转、放、免”力度，年内再取消下放一批，切实减少政府对微观事务的管理。该管的一定管到位，对规划、项目、政府性债务、长江岸线使用、山体水体、政府类信息化投资、安全生产和食品药品安全等，都建立有效的评价管理制度。该服务的切实服务好，进一步加大民生投入力度，提升为民服务水平；每月举办一次企业家圆桌会议，适时举办企业家沙龙，为实体经济提供全天候、全方位、全流程服务。

　　二是坚持科学决策。健全市政府工作规则，落实社情民意调查、公示听证、专家论证、风险评估、合法性审查、报告和协商、集体讨论决定等民主决策程序。完善城乡规划审查、政府信息公开和新闻发布制度。引入社会满意度评价，提高政府工作的透明度和公民参与度。

　　三是严格依法行政。自觉依照法定权限行使权力，认真接受人民代表大会及其常委会的法律监督和工作监督、人民政协的民主监督，以及社会公众监督和舆论监督。建立政府与人大、政协工作沟通的年初通气、年中通报、年底报告制度，每年确定一批议题，提请市人大监督推动和市政协民主协商。推进法治政府建设，切实加强规范性文件制订、行政复议和法律顾问工作。积极争取获批“较大的市”（28）。

　　四是强化能力提升。坚持政府系统领导干部每月集中学习制度，健全领导干部带头读书、带头思考、带头调研机制，提升战略思维和实践创新能力。加强公务员学习培训和挂职锻炼。开展第二轮公务员集中军训，进一步增强公务人员的大局意识、执行意识和责任意识。

　　五是保持清正廉洁。严格执行中央“八项规定”和省、市委“十项规定”，认真开展党的群众路线教育实践活动，持之以恒抓好“四风”（29）整改，扎实有效开展领导干部“三解三促”活动。突出抓好财政预决算公开，一律不新建政府性的楼堂馆所，财政供养的人员只减不增，公费接待、公费出国、公费购车只减不增。强化行政监察和审计监督，严格工程招投标、土地转让、政府采购等重点领域监管，从源头上预防和治理腐败。

　　各位代表，镇江正处于新的发展阶段，改革的征程已经开启，发展的号角催人奋进。让我们在“中国梦”的激励和感召下，在中共镇江市委的坚强领导下，与全市人民一道，以更加饱满的热情、更加昂扬的斗志、更加务实的作风，向着建设令人向往的现代化山水花园城市目标，奋勇前进！
